# Full-field 3D shape measurement of discontinuous specular objects by direct phase measuring deflectometry

**DOI:** 10.1038/s41598-017-11014-5

**Published:** 2017-08-31

**Authors:** Yue Liu, Shujun Huang, Zonghua Zhang, Nan Gao, Feng Gao, Xiangqian Jiang

**Affiliations:** 1 School of Mechanical Engineering, Hebei University of Technology, Tianjin, 300130 China; 2 Centre for Precision Technologies, University of Huddersfield, Huddersfield, HD13DH UK

## Abstract

With the advent of intelligent manufacturing, phase measuring deflectometry (PMD) has been widely studied for the measurement of the three-dimensional (3D) shape of specular objects. However, existing PMDs cannot measure objects having discontinuous specular surfaces. This paper presents a new direct PMD (DPMD) method that measures the full-field 3D shape of complicated specular objects. A mathematical model is derived to directly relate an absolute phase map to depth data, instead of the gradient. Two relevant parameters are calibrated using a machine vision-based method. On the basis of the derived model, a full-field 3D measuring system was developed. The accuracy of the system was evaluated using a mirror with known positions along an accurate translating stage. The 3D shape of a monolithic multi-mirror array having multiple specular surfaces was measured. Experimental results show that the proposed DPMD method can obtain the full-field 3D shape of specular objects having isolated and/or discontinuous surfaces accurately and effectively.

## Introduction

With the development of the three-dimensional (3D) optical shape measurement technique, it has been widely applied in the fields of reverse engineering, biological recognition and digitalization of cultural relics, etc. However, most of the existing techniques are applied to measure objects with diffused surface. There are a large number of transparent, black, and reflective objects in industrial applications. The research of shape measurement for these objects is still in the early stage. The main methods of measuring the 3D shape of such objects use a coordinate measuring machine^1^ or change the surface characteristics by spraying paint, which destroys the surface properties and is unacceptable in many fields. With the advent of intelligent manufacturing, there are many specular objects in actual industrial fields. It is therefore important to study optical 3D shape measurement methods for specular objects^2^.

Phase measuring deflectometry (PMD) or fringe reflection deflectometry (FRD) has been widely studied to test specular free-form surfaces^3, 4^ because of the advantages of non-contact operation, full-field measurement, fast acquisition, high precision and automatic data processing^5–7^. PMD has been applied to measure an aspheric mirror and dynamic specular surface and to detect subsurface cracking^8^, from a micro-size to a large specular surface^9–14^. Such a technique needs to display straight sinusoidal fringe patterns on a screen or to project a structured pattern onto ground glass. From a different viewpoint, fringe patterns reflected by a measured surface appear deformed with regard to the slope variation of the specular surface and the modulated fringe patterns can be captured by an imaging device, such as a charge-coupled device (CCD) camera. Phase information in the deformed fringe patterns is demodulated to obtain the slope of the measured specular surface and the 3D shape of the tested surface can then be reconstructed by integrating the gradients^15, 16^. The above PMD methods therefore only measure the local slope of smooth surfaces^17^, instead of the actual 3D shape. Owing to the integration procedure, complicated specular components having isolated and/or discontinuous surfaces^18^ cannot be directly measured from phase data. White light interferometry^19^, wavelength scanning interferometer^20^ and multiple wavelength interferometer^21^ can be used to measure specular objects having discontinuous surfaces. However, the field of view of the objective lens, the range of the scanner and the limited synthetic wavelength are restricted to the measurement range. These kind of interferometry are normally used for surface finish measurement instead of surface form measurement.

To solve the above problem, an FRP method has been used to measure absolute coordinates of discontinuous specular surfaces^22^. However, this method requires complicated spatial geometry computation. Moreover, the distance translated by a translating stage strongly affects the measurement accuracy, especially when the axis of translation and surface normal of the reference plane are not parallel. A zonal wavefront reconstruction algorithm has been implemented to realize three-dimensional, highly reflected and specular surface reconstruction^23^. Although gauge blocks with two different heights were tested, it is difficult to measure multiple discontinuous surfaces because this method needs each measured surface of the step object to have one perturbed stripe.

This paper presents a new direct PMD (DPMD) method that measures the full-field 3D shape of specular objects having isolated and/or discontinuous surfaces by directly building the relationship between the absolute phase map and depth data^24^. When a liquid crystal display (LCD) screen is located at two known positions along the normal direction, a mathematical model is derived to directly relate the phase to depth, instead of the slope of the measured specular surface. A plate beam splitter (BS) is used to realize the parallel design of two LCD screens and thus avoid mechanically moving a screen to two positions. Sinusoidal fringe patterns having optimum fringe numbers are generated by software and displayed on the two screens^25^. A CCD camera captures the two sets of deformed fringe pattern images from a reflected viewpoint. Wrapped and absolute phase maps are calculated using a four-step phase-shifting algorithm^26^ and an optimum three-fringe number selection method, respectively. After calibrating the system, depth information of a specular object can be directly obtained from the calculated phase map. Experimental results for a monolithic multi-mirror array having multiple specular surfaces show that the proposed measurement method can obtain the full-field 3D shape of specular objects with isolated and/or discontinuous surfaces accurately and effectively.

## Results

**Figure 1:**
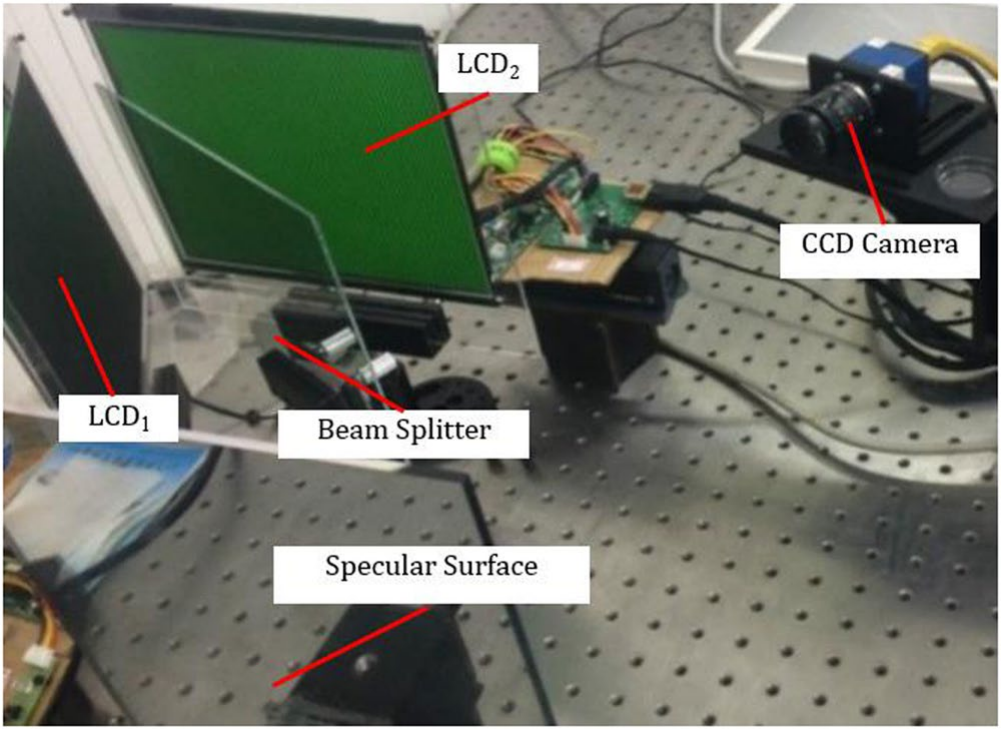
Hardware of the developed 3D measurement system.

A measurement system, as illustrated in Fig. 1, was developed to obtain the 3D shape of specular objects by displaying the same fringe pattern onto two LCD screens. The hardware system consists of a computer, a CCD camera, two LCD screens and a plane BS. The two LCD screens are from LG Electronics Inc. The CCD camera is the latest industrial camera from SVS Company with the model number ECO655 and resolution of 2050 × 2448 pixels. The camera supports external and internal trigger modes. The relative positions of the two LCDs and BS were adjusted to let LCD_2_ be parallel to the virtual image LCD_1_′ of LCD_1_. Using the proposed calibration methods, the two parameters d and ∆d were calibrated and their values are 154.532 mm and 17.905 mm. After the parameters of ∆d and d were calibrated using the method described above, the 3D shape of the specular surface could be measured by the developed system.

To evaluate the accuracy of the developed 3D measurement system, the plate mirror M_1_ was placed on an accurate translating stage with resolution of 1 μm. The plate was positioned at −3.7, −1.3, 1.3 and 3.7 mm with respect to the reference plane. At each position, depth data were calculated using the calibrated parameters. The translating distance of the stage, the measured average distance and absolute error (i.e., absolute difference between the measured average distance and position of the stage) are listed in Table 1. The maximum absolute error was smaller than 0.064 mm. The results clearly show that the proposed calibration method accurately converts the absolute phase into depth data. Because all the points on one plane mirror have the same depth value to the reference plane in principle, the flatness of the plane can be used to calculate the repeatability of the measurement in the same condition. The repeatability value of the measurement is 0.032 mm by measuring a mirror plane 10 times in the same condition.

**Table 1:** Experimental results for the accurately positioned mirror (Unit: mm).

Position of the mirror	−3.7	−1.3	1.3	3.7
Measured distance	−3.764	−1.353	1.341	3.743
Absolute error	0.064	0.053	0.041	0.043

**Figure 2:**
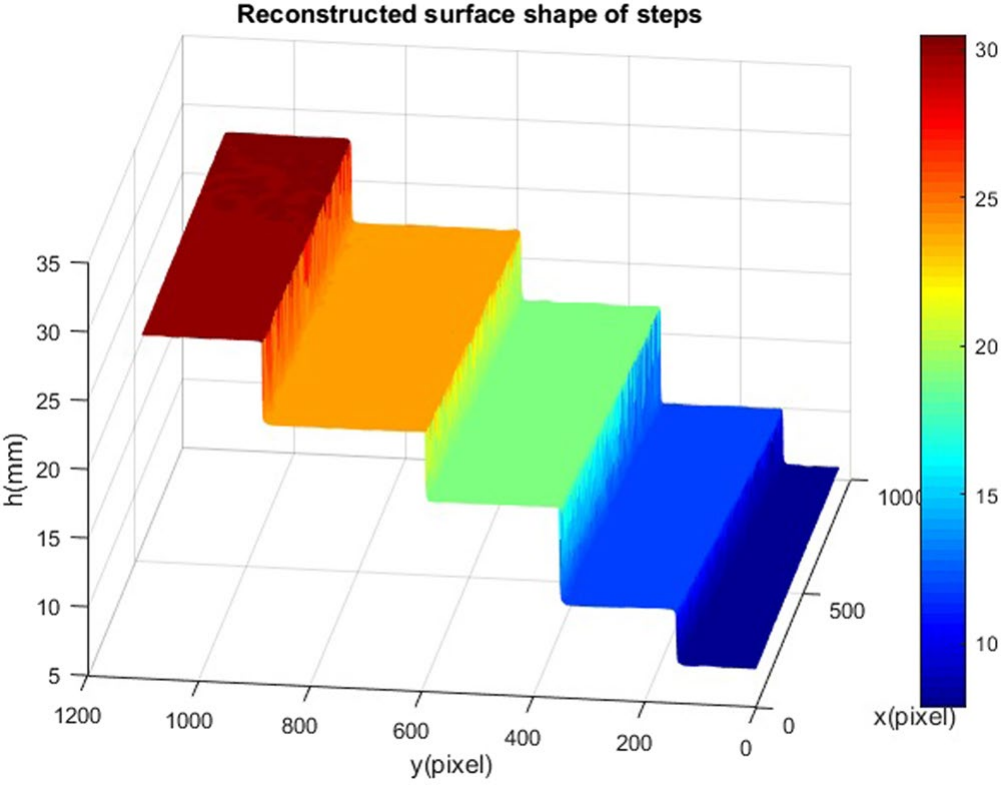
Measured depth of the measured step.

In order to further test the accuracy of the 3D measuring system, an artificial step having discontinuous specular surfaces has been designed and manufactured. The distance between neighboring steps was measured by a coordinate measuring machine (CMM). Twelve sinusoidal fringe patterns having optimum fringe numbers^25^ of 64, 63 and 56 were generated by software and sequentially displayed on the two LCD screens. The fringe patterns reflected by the specular surface of the artificial step were captured by the CCD camera. Depth data were obtained from the captured fringe patterns by using equation (5), as illustrated in Fig. 2. As listed in Table 2, the measured results by the deflectometry system were compared to that by the CMM method. The maximum absolute error is 0.052 mm.

**Table 2:** Experimental results on the measured step (Unit: mm).

Step distance	Measured distance	Absolute error
3.987	4.036	0.049
7.025	7.062	0.037
5.006	5.058	0.052
6.099	6.143	0.044

**Figure 3:**
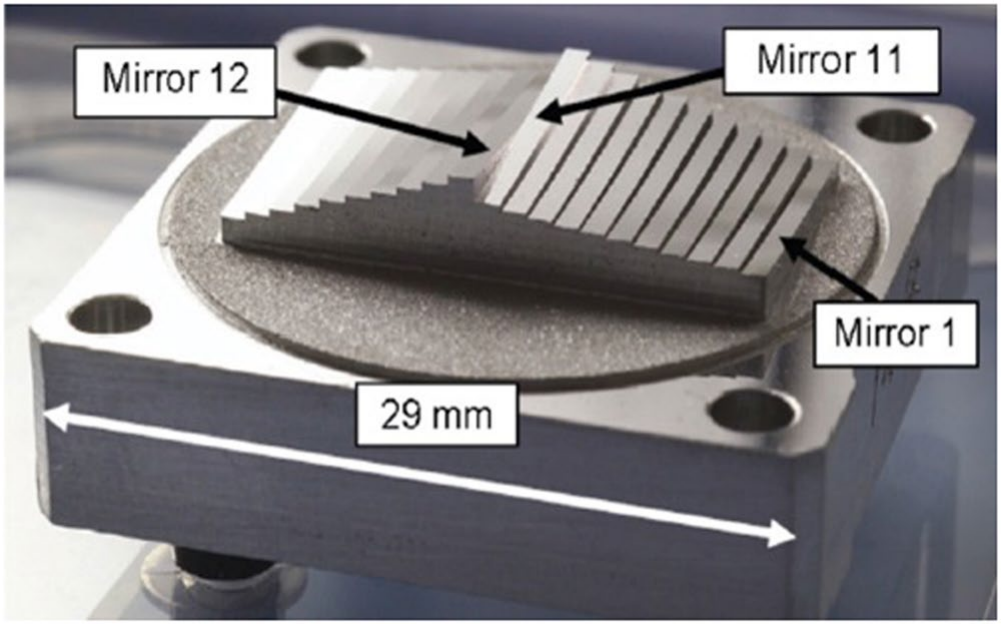
A monolithic multi-mirror array on the MIRI Spectrometer Optics for the James Webb Space Telescope.

**Figure 4:**
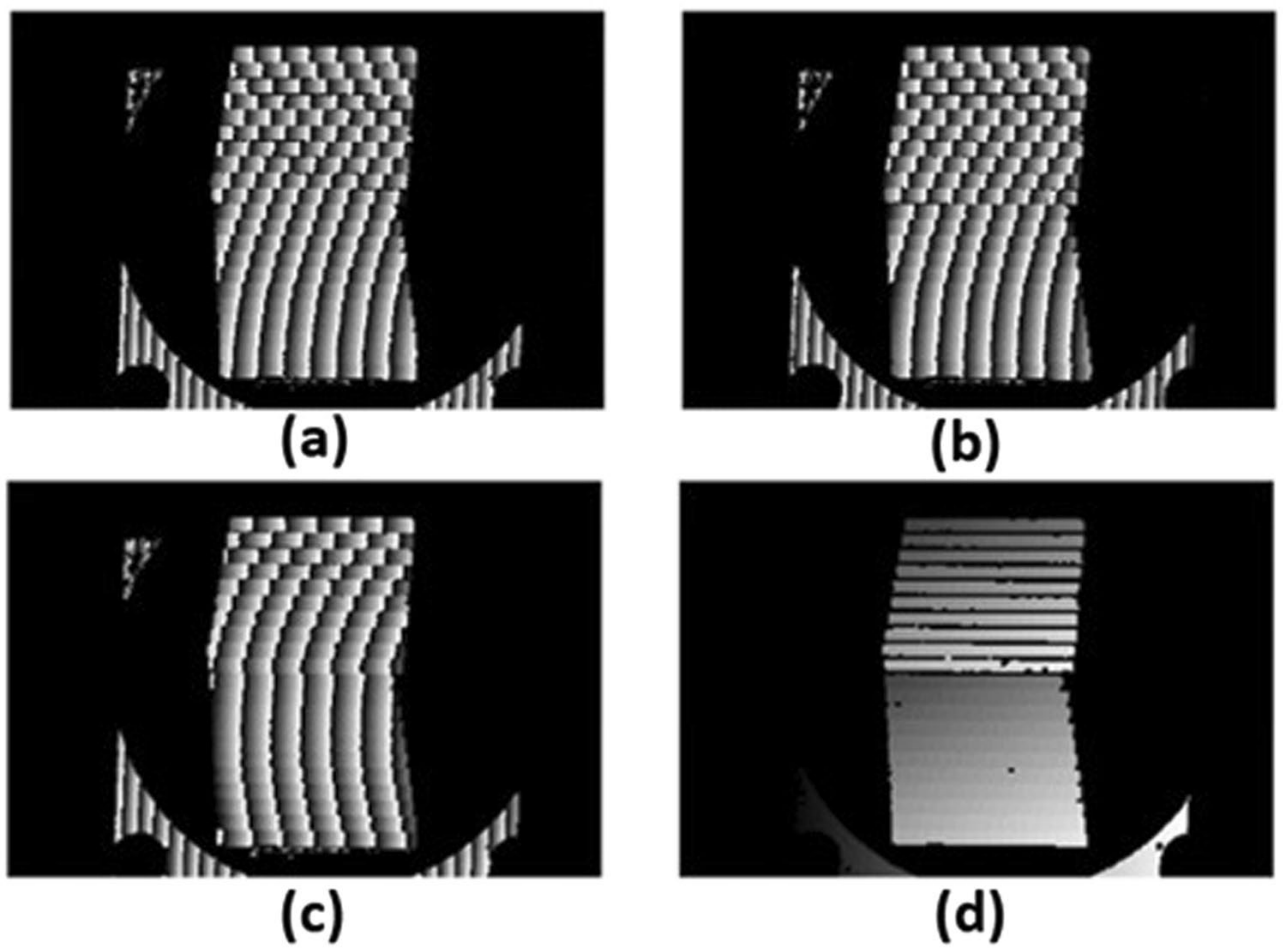
Phase of the monolithic multi-mirror array. (**a**–**c**) are three wrapped phase maps having 64, 63 and 56 projected fringes, while (**d**) is the absolute phase map.

**Figure 5:**
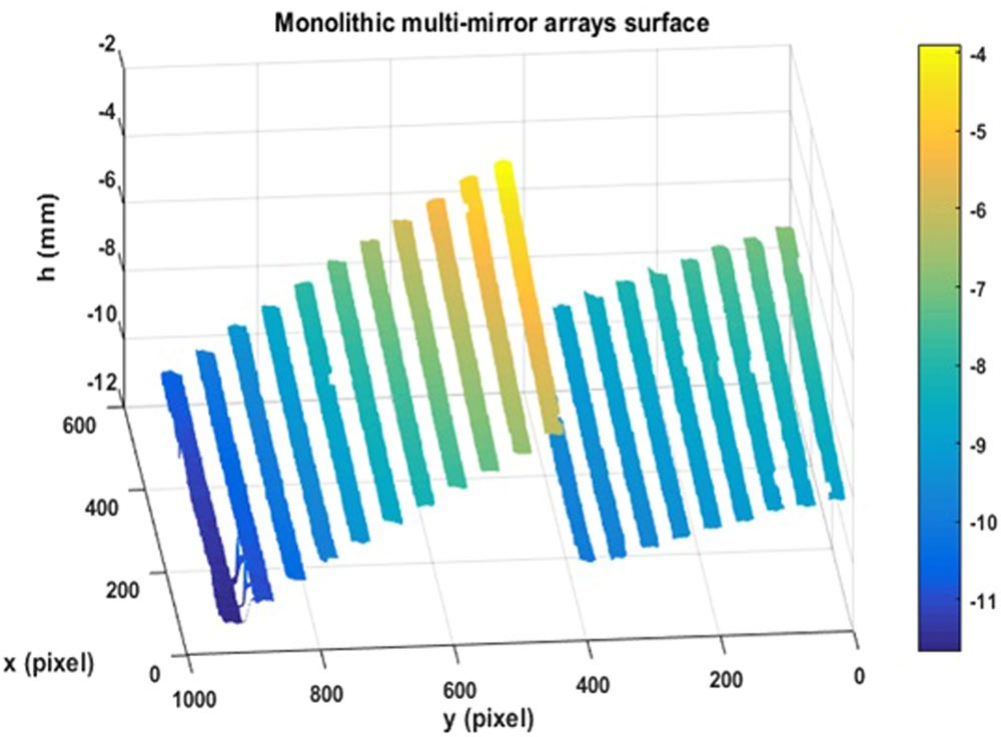
Measured depth of the monolithic multi-mirror array.

A monolithic multi-mirror array on the Mid-Infrared Instrument (MIRI) Spectrometer Optics for the James Webb Space Telescope, which has multiple discontinuous specular surfaces, was measured by the developed 3D measurement system, as illustrated in Fig. 3. Twelve sinusoidal fringe patterns having optimum fringe numbers of 64, 63 and 56 were generated by software and sequentially displayed on the two LCD screens. The fringe patterns reflected by the specular surface of the monolithic multi-mirror array were deformed and captured by the triggered CCD camera from another viewpoint. Using the four-step phase-shift algorithm, three wrapped phase maps were calculated for the measured specular object, as shown in Fig. 4(a)–(c). The absolute phase of each pixel was determined using the optimum three-fringe selection method, as shown in Fig. 4(d). Using the calibrated parameters ∆d and d, depth data were obtained as shown in Fig. 5. Because the reflected fringe patterns by the connecting surface cannot be seen by the CCD camera, only step surface data without the connecting surfaces are obtained. The results show that the proposed method can directly measure a specular object having isolated and/or discontinuous surfaces. In order to capture the reflected fringe patterns by the multiple surfaces, the base surface of the monolithic multi-mirror arrays needs to have an angle with respect to the reference plane. Therefore, the calculated depth data on both two groups (Mirror 1 to Mirror 11 and Mirror 12 to Mirror 21) have a positive slope.

## Discussion

This paper presented a new full-field 3D shape measurement method for specular surfaces that uses the direct relationship between absolute phase and depth. Two LCD screens and one BS were used to realize the design of two parallel screens. The same fringe pattern sets were displayed on the two screens and reflected by the specular surfaces of the measured objects. Two absolute phase maps were obtained from the captured fringe patterns using a four-step phase-shifting algorithm and an optimum three-fringe number selection method. After two parameters of the system were calibrated using a machine vision method, depth data could be directly derived from the obtained absolute phase map. Because depth directly relates to the absolute phase without needing gradient integration, the proposed method can measure specular objects having isolated and/or discontinuous surfaces. Initial experimental results on measuring the monolithic multi-mirror array on the MIRI Spectrometer Optics for the James Webb Space Telescope having multiple specular surfaces show that the developed 3D measurement system obtains depth data effectively and accurately.

The proposed DPMD method has the following advantages. (1) Ability of full-field measuring 3D shape of specular objects having isolated and/or discontinuous surfaces by using the direct relationship between an absolute phase map and depth data. (2) Simple operation during calibration and measurement due to no moving parts. (3) Potential high accuracy without error accumulation from gradient integration.

There are several future research directions for the proposed method. (1) Capturing speed: The generated fringe patterns will be coded into different color channels of the two LCD screens and then captured simultaneously by the corresponding color channels of a color CCD camera. (2) Accuracy: Efficient calibration methods will be applied to obtain more accurate parameters and the performance will be evaluated using other optical metrology. (3) Calibration: Only the relationship between the absolute phase and depth was determined. It is beyond the scope of this paper with regard to pixel position to transverse coordinate values. (4) Error analysis: Effects of all kinds of error sources on the measurement results need to be analyzed; e.g., non-parallelism of the two screens and inaccuracy of the calibrated parameters ∆d and d.

## Methods

**Figure 6:**
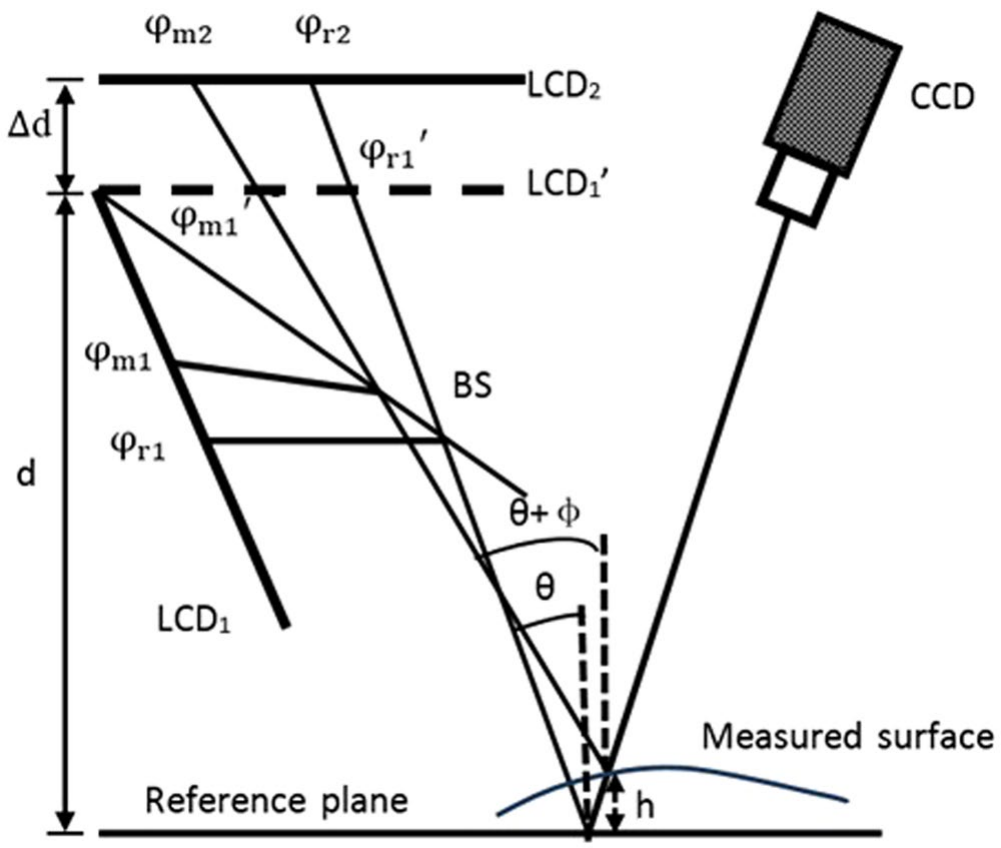
Schematic setup of the 3D measurement system.

Figure 6 shows the schematic setup of the proposed 3D shape measurement system of specular surfaces using DPMD. The system comprises two LCD screens, a CCD camera and a plate BS.

The BS is set in one proper position, such that a virtual image LCD_1_′ of LCD_1_ is parallel to LCD_2_, just like two LCD screens are located at two different positions. Moreover, the two screens are parallel to the reference plane. The parallelism is established by repeating adjustment of the relative orientations of the three components (the two LCD screens and the mirror at reference plane) and generating pre-distorted fringe patterns for compensation. Hollow ring markers matrix is generated in software and displayed on both LCD screens. The CCD camera captures the hollow ring markers matrix on three components surface. The external parameters of the three components in camera coordinate system are obtained by using the known separation between neighboring markers and the internal parameters of the camera. Based on the obtained external parameters, the relative orientation of the three components is adjusted. This procedure is repeated several times until the three components are nearly parallel. Finally, a software-based method is applied to generate pre-distorted fringe patterns to compensate for the non-parallelism of the three components^27^. Therefore, the two LCD screens and the mirror at the reference plane are parallel. The plate beam splitter is therefore used to realize the parallel design of the two screens to avoid mechanically moving a screen to two positions LCD_1_′ and LCD_2_ along the normal of the reference plane. The layout has two advantages. (1) There are no moving parts during calibration and measurement, and there is thus no effect on the resultant data from moving components. (2) Two sets of fringe patterns can be captured simultaneously by a color CCD camera when the fringe patterns are coded into different color channels of the two LCD screens.

The distance between LCD_1_′ and LCD_2_ and that between the reference plane and LCD_1_′ are denoted ∆d and d, respectively. When the same sinusoidal fringe patterns having the optimum fringe numbers are generated by software and displayed on the two LCD screens, they are reflected and deformed by the reference mirror and the specular surface under test. The deformed fringe patterns are captured by the CCD camera from another viewpoint for post-processing. The corresponding absolute phase of each point on the two LCD screens can be calculated by combining the four-step phase-shifting algorithm and the optimum three-fringe number selection method.

Assuming that the imaging system is a pinhole projection, two rays of light are displayed and reflected into the CCD camera via the measured surface and the reference mirror, as illustrated in Fig. 5. The two incident rays correspond to the same reflection light. The absolute phases of the two incident rays are denoted φ_r1_ (or ${{\rm{\phi }}}_{{\rm{r}}1}^{^{\prime} }$) and φ_*r*2_ on the reference mirror and φ_m1_ (or ${{\rm{\phi }}}_{{\rm{m}}1}^{\prime}$) and φ_*m*2_ on the measured specular surface. θ and θ + Φ are the angle between the incident ray and the normal vector of the reference mirror and the angle between the incident ray and the normal vector of the measured specular surface, respectively. The period of the displayed fringe pattern on the LCD screen is denoted q. ∆l is the distance on LCD_1_′ between the two incident rays because of the height and gradient of the measured surface. Parameter h stands for the height of the measured specular surface with respect to the reference mirror.

The geometric relationship in Fig. 6 gives the relations

1 $$({{\rm{\phi }}}_{{\rm{r}}1}-{{\rm{\phi }}}_{{\rm{r}}2}){\rm{q}}/2{\rm{\pi }}={\rm{\Delta }}\text{dtan}\,{\rm{\theta }},$$

2 $$({{\rm{\phi }}}_{{\rm{m}}1}-{{\rm{\phi }}}_{{\rm{m}}2}){\rm{q}}/2{\rm{\pi }}={\rm{\Delta }}\text{dtan}({\rm{\theta }}\,+{\rm{\Phi }}),$$

3 $$({\rm{d}}\,+{\rm{h}})\tan \,{\rm{\theta }}+{\rm{\Delta }}l=({\rm{d}}-{\rm{h}})\tan \,({\rm{\theta }}+{\rm{\Phi }}),$$

4 $${({\rm{\phi }}}_{{\rm{r}}1}-{{\rm{\phi }}}_{{\rm{m}}1}){\rm{q}}/2{\rm{\pi }}={\rm{\Delta }}{\rm{l}}.$$

From equations (1)–(4), height h of the measured specular surface is

5 $${\rm{h}}=\frac{{\rm{\Delta }}{\rm{d}}({{\rm{\phi }}}_{{\rm{r}}1}-{{\rm{\phi }}}_{{\rm{m}}1})-{\rm{d}}[({{\rm{\phi }}}_{{\rm{r}}1}-{{\rm{\phi }}}_{{\rm{r}}2})-({{\rm{\phi }}}_{{\rm{m}}1}-{{\rm{\phi }}}_{{\rm{m}}2})\,]\,}{({{\rm{\phi }}}_{{\rm{m}}1}-{{\rm{\phi }}}_{{\rm{m}}2})+({{\rm{\phi }}}_{{\rm{r}}1}-{{\rm{\phi }}}_{{\rm{r}}2})}.$$

This equation clearly shows that height information can be directly calculated from the captured fringe patterns on the measured specular surface only if the two parameters d and ∆d and phase information on the reference plane are known beforehand. Because the optimum three-fringe number selection method is used to calculate the absolute phase pixel by pixel, the specular objects having isolated and/or discontinuous surfaces can be measured employing the proposed method.

**Figure 7:**
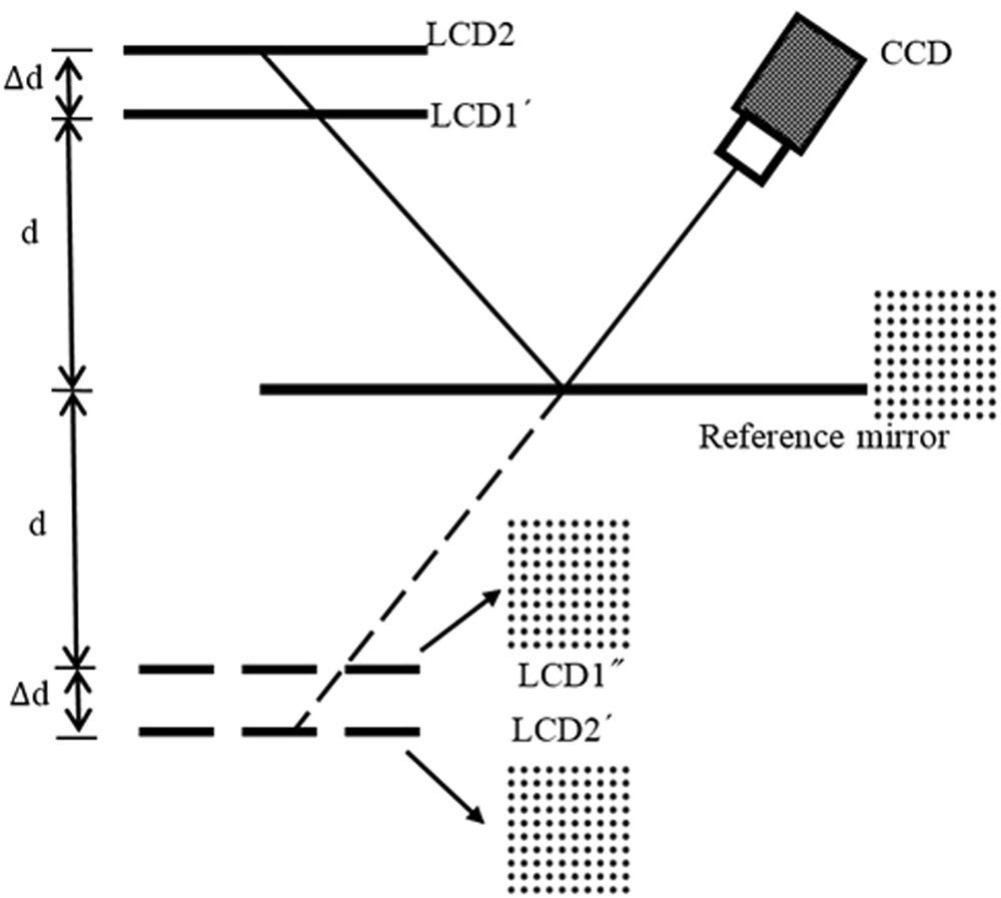
Diagram of the calibration of ∆d.

To obtain 3D shape data, an important step is to build the relationship between the absolute phase and depth. According to the derived mathematical model expressed as equation (5), the parameters of ∆d and d need to be determined beforehand. Two accurate plane mirrors were manufactured. M_1_ has an ideal plane surface while M_2_ has a hollow ring marker matrix with known separation. M_1_ is fixed on an accurate translating stage and moved to a known position ∆h along the normal direction of the two LCD screens, as illustrated in Fig.7. At each mirror position, fringe pattern sets having the optimum fringe numbers are generated and displayed on LCD_2_ and LCD_1_. The displayed fringe patterns are reflected by M_1_ and captured by the CCD camera.

The geometric relationship in Fig.7 gives the relations

6 $$({{\rm{\phi }}}_{{\rm{r}}1}^{\prime} -{{\rm{\phi }}}_{{\rm{r}}2})\frac{{\rm{q}}}{2{\rm{\pi }}}={\rm{\Delta }}\text{dtan}\,{\rm{\theta }},$$

7 $$({\rm{d}}+{\rm{\Delta }}{\rm{h}})\tan \,{\rm{\theta }}-{\rm{\Delta }}{\rm{l}}=({\rm{d}}-{\rm{\Delta }}{\rm{h}})\tan \,{\rm{\theta }},$$

8 $${\rm{\Delta }}{\rm{l}}=({{\rm{\phi }}}_{{\rm{m}}1}^{\prime} -{{\rm{\phi }}}_{{\rm{r}}1}^{\prime} )\frac{{\rm{q}}}{2{\rm{\pi }}},$$

where the parameters q, d, ∆d, ∆l and θ have the same meaning as before. The obtained absolute phase has the relationship

9 $${\rm{\Delta }}{\rm{d}}=2{\rm{\Delta }}{\rm{h}}({{\rm{\phi }}}_{{\rm{r}}1}^{\prime} -{{\rm{\phi }}}_{{\rm{r}}2})/\,({{\rm{\phi }}}_{{\rm{m}}1}^{\prime} -{{\rm{\phi }}}_{{\rm{r}}1}^{\prime} ).$$

In principle, one known depth ∆h and the corresponding phase value can determine ∆d. To improve the accuracy of ∆d, the translating stage moves to several known positions to build an over-determined equation set.

**Figure 8:**
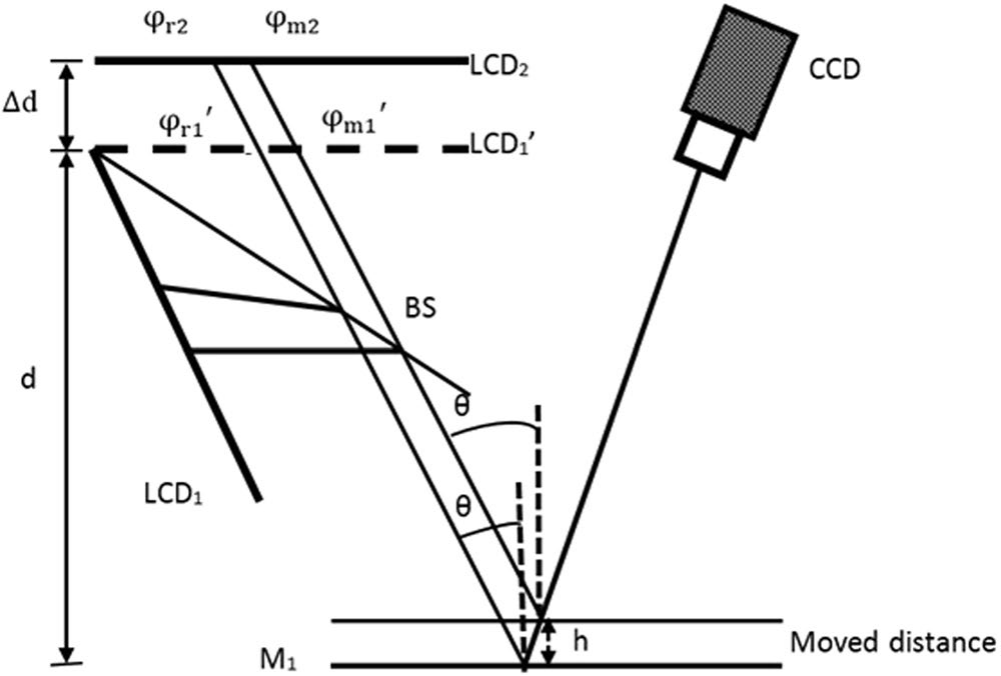
Diagram of the calibration of d.

To determine d, mirror M_2_ is positioned parallel to the LCD screen and its surface is chosen as the reference plane, as illustrated in Fig. 8. After calibrating the internal parameters of the CCD camera^28–30^, the orientation of M_2_ is determined in the camera coordinate system using the hollow ring marker matrix^31^ on the surface of M_2_. The same hollow ring marker matrix is generated by software and displayed on the LCD_1_ screen. Owing to the reflection from the surface of M_2_, the CCD camera can view and capture the marker matrix at the position LCD_1_″ (the virtual image of LCD_1_′). The orientation of LCD_1_″ (LCD_1_′) is therefore obtained in the camera coordinate system using the hollow ring marker matrix on LCD_1_^32^. Distance d can be calculated using the obtained orientation of M_2_ and LCD_1_ in the same camera coordinate system.
